# Adverse effects of microplastics and oxidative stress-induced MAPK/Nrf2 pathway-mediated defense mechanisms in the marine copepod *Paracyclopina nana*

**DOI:** 10.1038/srep41323

**Published:** 2017-01-24

**Authors:** Chang-Bum Jeong, Hye-Min Kang, Min-Chul Lee, Duck-Hyun Kim, Jeonghoon Han, Dae-Sik Hwang, Sami Souissi, Su-Jae Lee, Kyung-Hoon Shin, Heum Gi Park, Jae-Seong Lee

**Affiliations:** 1 Department of Biological Science, College of Science, Sungkyunkwan University, Suwon 16419, South Korea; 2 Department of Chemistry, College of Natural Sciences, Hanyang University, Seoul 04763, South Korea; 3 Univ. Lille, CNRS, Univ. Littoral Cote d’Opale, UMR 8187, LOG, Laboratoire d’Océanologie et de Géosciences, F 62930 Wimereux, France; 4 Department of Life Sciences, College of Natural Sciences, Hanyang University, Seoul 04763, South Korea; 5 Department of Marine Sciences and Convergent Technology, College of Science & Technology, Hanyang University, Ansan 15588, South Korea; 6 Department of Marine Resource Development, College of Life Sciences, Gangneung-Wonju National University, Gangneung 25457, South Korea

## Abstract

Microplastic pollution causes a major concern in the marine environment due to their worldwide distribution, persistence, and adverse effects of these pollutants in the marine ecosystem. Despite its global presence, there is still a lack of information on the effect of microplastics on marine organisms at the molecular level. Herein we demonstrated ingestion and egestion of nano- (0.05 μm) and micro-sized (0.5 and 6 μm) polystyrene microbeads in the marine copepod *Paracyclopina nana*, and examined molecular responses to exposure to microbeads with *in vivo* endpoints such as growth rate and fecundity. Also, we proposed an adverse outcome pathway for microplastic exposure that covers molecular and individual levels. This study provides the first insight into the mode of action in terms of microplastic-induced oxidative stress and related signaling pathways in *P. nana*.

Pollution by plastics is growing concern in aquatic environments, since approximately 299 million tons of plastics are were produced from the 1950 s to 2013 (Plastics Europe, 2015)^1^. At least 10% of plastics produced are discarded into the marine environment^2^,^3^ with an estimated >5 trillion pieces of plastic debris polluting worldwide oceans including surface water, seafloors, estuaries, and shorelines^4^.

The adverse impacts of ocean microplastics on the aquatic ecosystem are well supported by research^5^. In the marine environment, plastic debris are progressively broken into small fragments referred as microplastics (<5 mm) in response to wave action, ultraviolet (UV) radiation, and hydrolysis and also into nano-sized particles^6^,^7^,^8^. These small plastics have a long degradation time and high bioavailability due to their high volume-to-surface ratio^9^,^10^,^11^. The size of the plastic particle is considered as the most significant factor, causing adverse effects on marine invertebrates. For example, the size-dependent toxicity of microplastics has been observed in the rotifer *Brachionus koreanus*^12^. In the copepod *Tigriopus japonicus*^13^, exposure to 0.05- and 0.5-μm polystyrene microbeads led to significant retardation of developmental time and decreased survival rate, while 6-μm polystyrene microbeads exposure did not lead to significant growth retardation.

Ingestion of microplastics is a common phenomenon in marine biota from low-trophic level organisms to predators such as the fur seal *Arctocephalus* sp.^14^, the whales *Megaptera novaeangliae* and *Mesoplodon mirus*^15^,^16^, and the zebrafish *Danio rerio*, the Japanese medaka *Oryzias latipes* and several species of pelagic and demersal fish^17^,^18^,^19^. Zooplankton also ingest microplastics as primary consumers including the copepod *T. japonicus*^13^,^20^,^21^,^22^, the water flea *Daphnia*^23^, and the rotifers^12^,^24^ which can have negative effects on survival and reproduction.

Zooplankton play an important role in the aquatic food chain as a primary consumer and a major food source for higher trophic organisms^25^,^26^,^27^. Thus, ingestion of microplastics by zooplankton could have critical impacts on the aquatic ecosystemas as a whole. Among zooplankton, copepods are a popular model for ecotoxicological studies, as copepods play an important role in the aquatic ecosystem and are sensitive to environmental stressors with several advantages with regard to laboratory experiments such as small body size (~1 mm), a short life cycle (~2 weeks), easy culture, and a high reproduction rate^28^. Moreover, copepods are susceptible to ingested microplastics^13^,^21^,^22^,^29^. Based on these favorable characteristics of copepods, the cyclopoid copepod *Paracyclopina nana* was chosen as the experimental model in the present study^30^.

Here, we investigated the effects of different sizes of microplastics (0.05 μm for nano-sized microplastics and 0.5 and 6 μm for micro-sized microplastics) in the copepod *P. nana* at molecular levels using *in vivo* endpoints. *In vivo* endpoints were measured to identify the consequences of size-dependent microplastic toxicity on *P. nana*. Also, the activation of mitogen-activated protein kinase (MAPK) and nuclear factor erythroid 2-related factor 2 (Nrf2), which acts as a downstream transcription factor of MAPKs, was studied along with the reactive oxygen species (ROS) levels, to examine whether they are involved in signal transduction pathways, which may lead to the activation of oxidative stress-induced defense system in *P. nana*. This study will provide a better understanding of how the copepod *P. nana* manages microplastic-induced oxidative stress via MAPK/Nrf2 pathways with regard to the adverse outcome pathway (AOP).

## Results

### Ingestion and egestion of polystyrene microbeads

**Figure 1:**
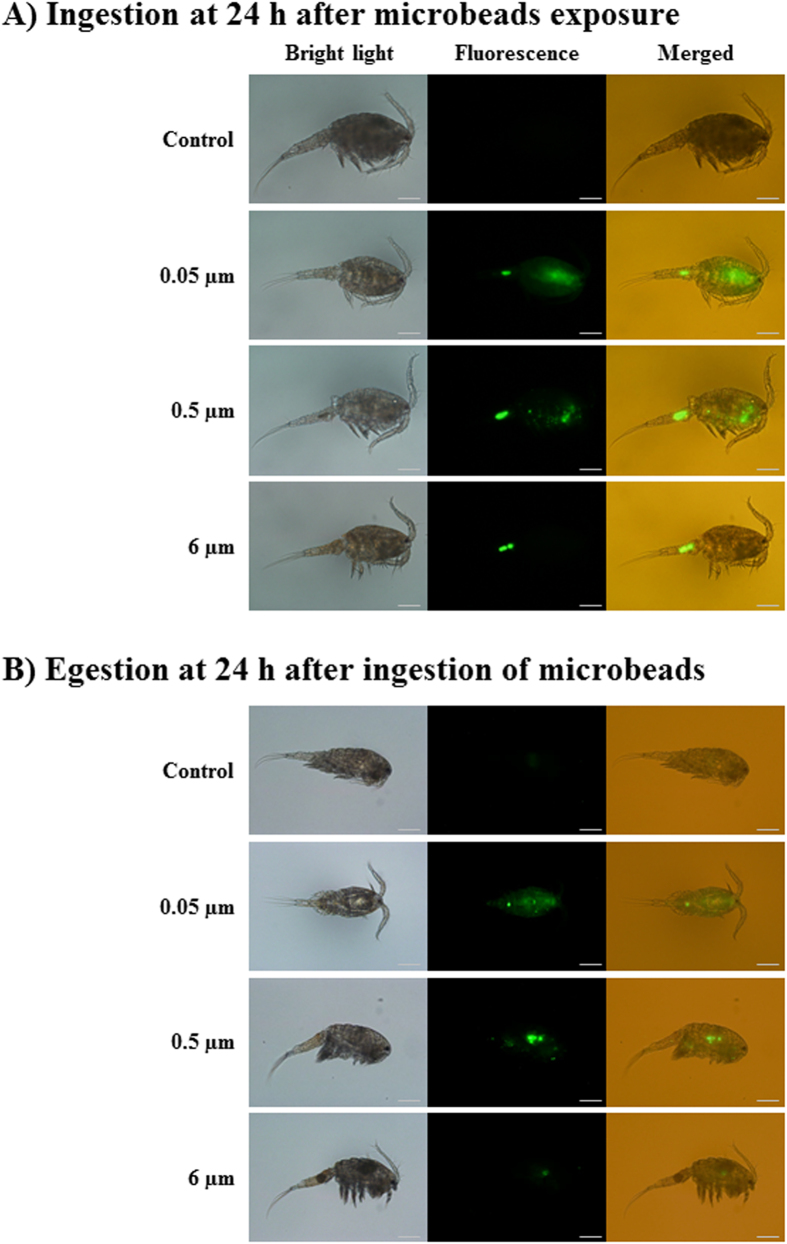
Images of fluorescently labeled polystyrene microbeads of different diameters ingested by *P. nana*: (**A**) Ingestion and (**B**) Egestion. Ingestion images were taken of copepods exposed to fluorescently labeled microbeads at 10 μg/ml for 24 h. Images for egestion were taken of copepods that were transferred into clean ASW and incubated for 24 h after ingestion. The scale bar indicates 200 μm.

Ingestion and egestion of microplastics in *P. nana* were demonstrated with different sizes of fluorescently-labeled microbeads (0.05, 0.5, and 6 μm). Microbeads of all sizes were ingested by *P. nana* as shown via fluorescence in the body of organisms but 0.05 μm microbeads were widely retained in the body (Fig. 1A).

Egestion of fluorescently-labeled microbeads varied depending on microbead sizes. Fluorescent 0.05- and 0.5-μm microbeads were observed until 24 h post-ingestion, while 6-μm microbeads had disappeared (Fig. 1B).

### *In vivo* effects of polystyrene microbeads exposure

**Figure 2:**
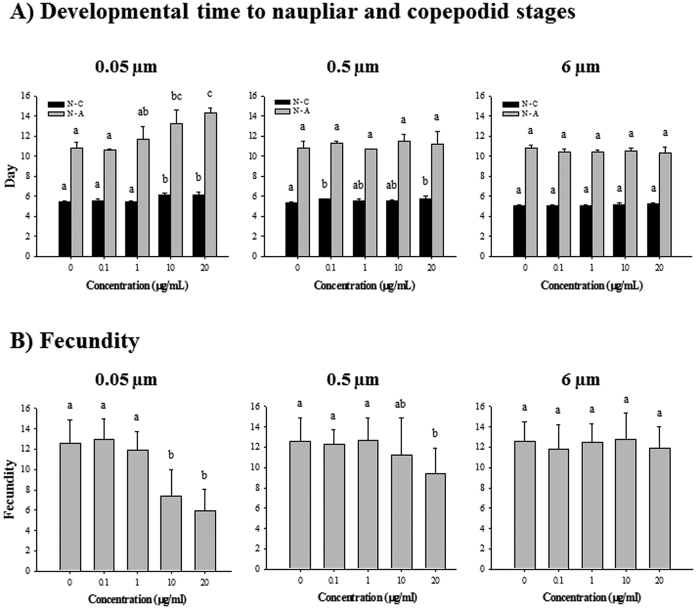
Effects of exposure to polystyrene microbeads of different diameters (0.05, 0.5, and 6 μm) on *P. nana*: (**A**) growth rate, (**B**) fecundity. Copepods were exposed to microbeads (0.1, 1, 10, and 20 μg/mL) for 24 h. The black bar (N-C) indicates the time taken from nauplius to copepodid stage, whereas the grey bar (N-A) shows the time from nauplius to adult stage. Differences between groups were analyzed for significance using Tukey’s multiple comparison test. Different letters above columns indicate significant differences (*P* < 0.05).

*P. nana* exposed to microbeads with a 0.05-μm diameter resulted in developmental delays and reduced in fecundity in *P. nana* in a dose-dependent manner, while 0.5-μm microbeads-exposed *P. nana* led to delayed molting (nauplii to copepodid) (*P* < 0.05) without a significant retardation in overall development. In the case of *P. nana* exposed to 6-μm microbeads, there were no observable *in vivo* effects (Fig. 2A,B).

### Levels of ROS and phosphorylation of MAPKs

**Figure 3:**
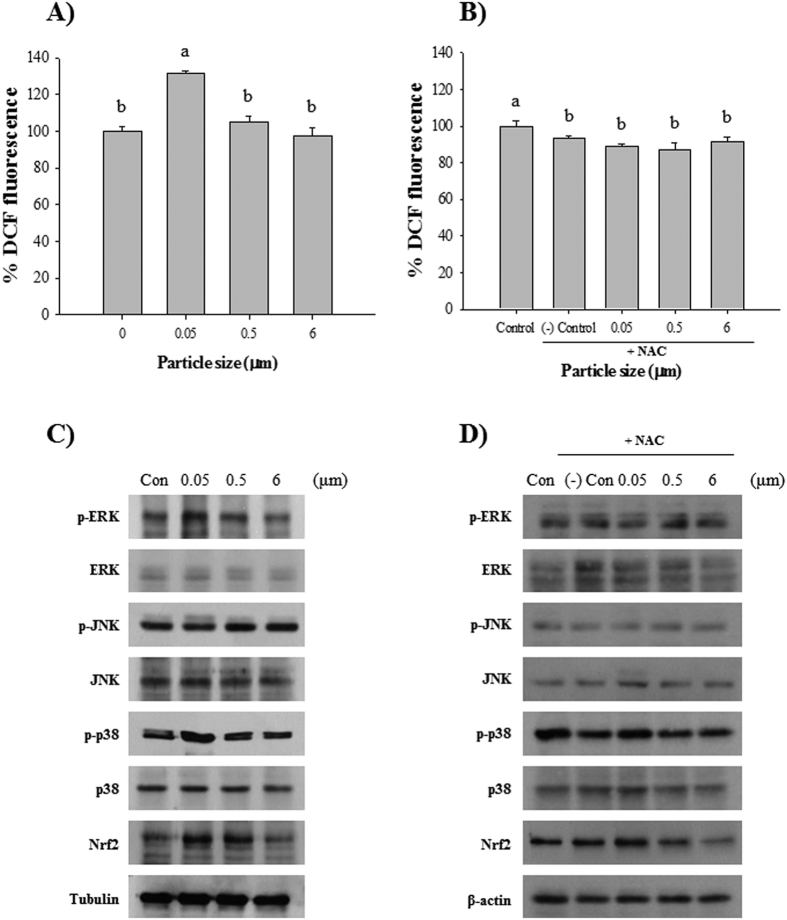
Effects of exposure to polystyrene microbeads of different diameters (0.05, 0.5, and 6 μm) on ROS production and phosphorylation of MAPK signaling proteins: (**A**) ROS levels, (**B**) ROS levels with NAC treatment (0.5 mM), (**C**) phosphorylation of MAPK proteins, and (**D**) phosphorylation of MAPK proteins with NAC treatment (0.5 mM). All polystyrene microbeads were administerd at 20 μg/ml. Levels of ROS are represented as % of controls. Different letters above columns indicate significant differences, defined as *P* < 0.05.

Only *P. nana* exposed to 0.05-μm microbeads exhibited a significant increase (*P* < 0.05) in intracellular ROS level, while organisms exposed to 0.5- and 6-μm microbeads were similar levels to the control group after 24 h exposure (20 μg/mL) (Fig. 3A). To investigate whether microplastic-induced ROS are a trigger for oxidative stress in *P. nana,* N-acetyl-L-cysteine (NAC; 0.5 mM) was co-administered with microbeads. Intracellular ROS were not generated by NAC treatment (Fig. 3B).

*P. nana* showed increased phosphorylation of extracellular signal-regulated kinase (p-ERK) and p38 (p-p38) with Nrf2, indicating a positive correlation with intracellular ROS levels. In the case of phosphorylated c-Jun N-terminal kinase (p-JNK), there was no difference in phosphorylation status (Fig. 3C). To investigate whether microplastic-induced ROS are trigger for the activation of p-ERK, p-p38, and Nrf2, NAC (0.5 mM) was co-administered with microbeads. Phosphorylation of ERK and p38 with Nrf2 was prevented by NAC treatment (Fig. 3D).

**Figure 4:**
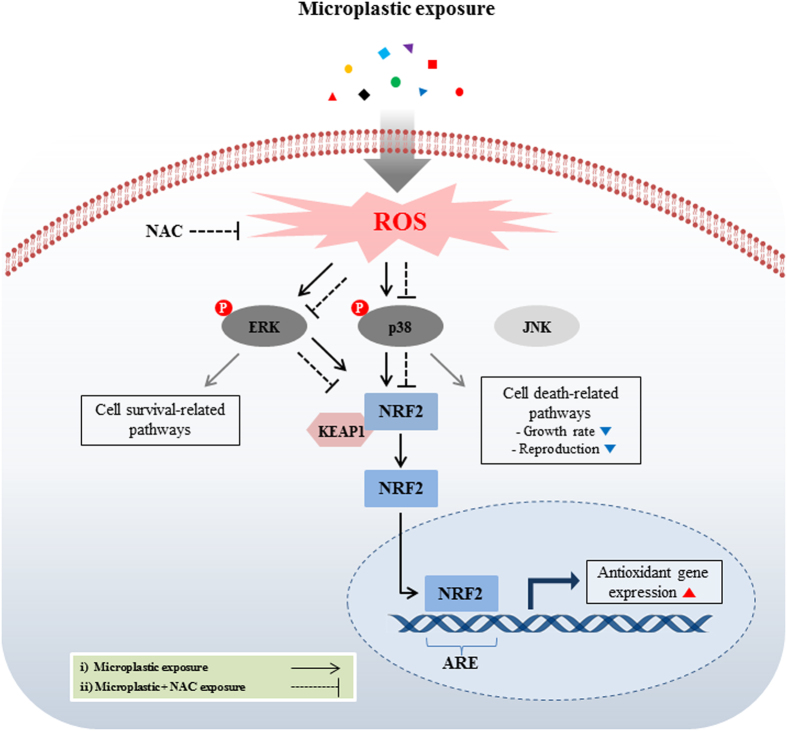
Schematic diagram of the suggested microplastic-mediated oxidative stress signaling pathway in *P. nana*. Elevation of intracellular ROS leads to activation of signal transductions mediated by p-EKR, p-P38, and subsequent Nrf2 activation. Nrf2 activation results in the induction of antioxidant genes, allowing cells to be protected against oxidative stress. Lines in black were drawn based on the result of the present study and lines in gray were drawn based on previous studies.

Based on data from this study, a schematic diagram of molecular response in *P. nana* in response to microplastic exposure was presented in Fig. 4.

### Antioxidant enzymatic activities of GPx, GR, GST, and SOD

**Figure 5:**
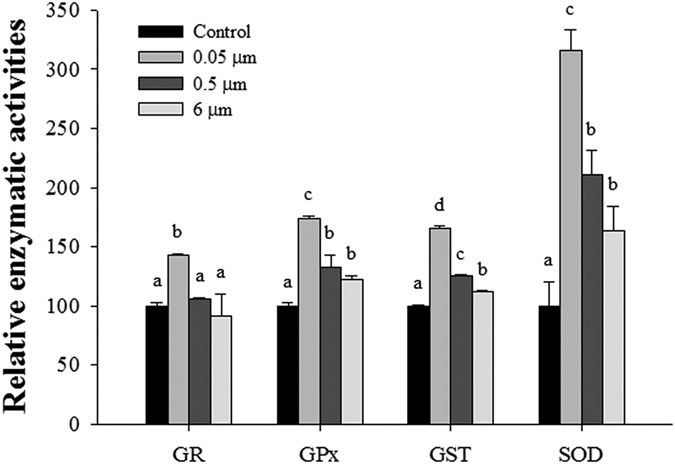
Effects of exposure to polystyrene microbeads of different diameters (0.05, 0.5, and 6 μm) on antioxidant-related enzymes. All polystyrene microbeads were used at 20 μg/ml. Enzymatic activity is presented as % of controls. Different letters above columns indicate significant differences, defined as *P* < 0.05.

To examine the defense mechanism in response to microplastic-induced oxidative stress in *P. nana*, antioxidant enzymatic activity was measured after 24 h exposure to 0.05-, 0.5-, and 6-μm microbeads (20 μg/mL). All antioxidant enzymes had the highest activity in animals exposed to 0.05-μm microbeads and followed by 0.5- and 6-μm microbeads (Fig. 5A).

## Discussion

Copepods are one of the most important species in aquatic ecosystems but have poor feeding selectivity, which can result in the ingestion of ambient microplastics^13^,^21^,^22^,^29^. For examples, in the copepod *Centropages typicus*, ingestion of polystyrene microbeads (0.4–30.6 μm) induced decrease in algal ingestion rate^22^. Also, in the copepod *Calanus helgolandicus*, algal ingestion, fecundity, and survival rate were negatively affected by ingestion of 20-μm polystyrene microbeads^21^. In the nano-sized and micro-sized microbeads-ingested copepod *T. japonicus*, their growth rate and survival rate was decreased^13^.

In *P. nana*, all different sizes of microbeads (0.05, 0.5, and 6 μm) were ingested. Interestingly, the 0.05-μm microbeads-exposed group had different fluorescence appearance compared to the 0.5- and 6-μm microbeads-exposed group. Fluorescence in 0.05-μm microbead-exposed *P. nana* was dispersed throughout the body, while florescence of 0.5- and 6-μm microbead-exposed *P. nana* was mostly limited to the digestive organs. Previously, molecular simulations revealed that nano-sized polystyrene is able to permeate biological membranes which may alter biological processes and cause cellular damage^31^. Moreover, nano-sized polystyrene microbeads with sizes ranging from 0.04 to 0.05 μm entered cells via an endocytosis pathway^32^. Taken together, the dispersed fluorescence observed in 0.05-μm microbead-exposed *P. nana* could be explained by translocation of polystyrene microbeads across the cellular membranes through the digestive organs of *P. nana*.

Compared to ingestion of microplastics, excretion of microplastics is not fully understood. In this study, 0.05-μm microbeads-exposed *P. nana* showed prolonged retention time in the body 24 h after ingestion compared to 0.5- and 6-μm microbeads, suggesting that larger microbeads are more easily egested compared to smaller microbeads. In the case of 0.05-μm microbeads, the dispersed fluorescence was seen througout the body of *P. nana* after 24-h exposure as seen in the rotifer *B. koreanus*^12^. In *B. koreanus*, 0.05-μm polystyrene microbeads were retained for a longer time periods in the digestive organs compared to that with 0.5- and 6-μm microbeads, causing more negative effects on lifespan, growth time, fecundity, and reproduction time. To date, only a few studies have explored the repercussions of microplastic retentions in aquatic organisms. For example, reproduction was negatively affected by prolonged exposure to polystyrene microbeads in the copepod *C. helgolandicus*^22^, while energy reserves were decreased with inflammatory responses in the lugworm *Arenicola marina*^33^. Taken together, prolonged retention of nano-sized microbeads in *P. nana* may have toxic risks related to the internalization of microbeads in cells, which could inhibit digestion, resulting in energy depletion and cell damage. Also, this kind of hindrance to obtaining food may lead to growth retardation and fecundity in nano-size microbead-exposed *P. nana*.

Apart from the prolonged retention of nano-sized microbeads and the subsequent effects on *P. nana*, ingestion of microbeads by copepods possibly could cause ecological issues due to their trophic level as a primary consumer and an important food source for higher consumers in the food chain^28^. Thus, bioaccumulation of microplastics could occur along with food chain. For example, 10-μm microbeads were transferred to the mysid shrimp *Neomysis integer* via ingested zooplankton^34^. Also, in the brine shrimp *Artemia* sp., microplastics, ranging from 1 to 20 μm in size, were accumulated and subsequently transferred to the zebrafish *D. rerio*^35^. Thus, microplastic ingestion could cause a considerable effect on key species in the aquatic ecosystem and not limited to an individual level. In this regard, ingestion of micro-sized microbeads could potentially cause negative effects on other organisms in the food web due to prey-predation relationships, although they had fewer *in vivo* effects at an individual level in *P. nana* compared to nano-sized microbeads.

Polystyrene microbead exposure led to adverse outcomes in the copepod *P. nana*. Particularly, 0.05- and 0.5-μm microbead-exposed *P. nana* had adverse effects on growth rate and fecundity, while there was no significant growth retardation (*P* < 0.05) in 6-μm microbead-exposed *P. nana* compared to controls. Several studies also supported the toxicity of nano-sized microbeads compared to larger microbeads in aquatic invertebrates. For example, in the rotifer *B. koreanus,* different sizes of polystyrene microbeads (0.05, 0.5, and 6 μm) caused a significant size-dependent decrease in growth rate, reproduction, lifespan, and body size^12^. In the copepod *T. japonicus*, growth and survival rates were also decreased according to size^13^. In the water flea *D. magna*, nano-sized polystyrene microbeads (~0.07 μm) exposure reduced both growth rate and body size^36^. Thus, the relationship between toxicity and particle size could be explained by the bioavailability of particles into aquatic organisms. Thus, it becomes more toxic as the particle decreases in size, since bioavailability increases with increasing surface-to-volume ratio^37^,^38^. Moreover, nano-sized polystyrene microbeads can permeate lipid membranes which can result in cellular damages^31^,^32^. Taken together, in *P. nana*, nano-sized polystyrene microbeads (0.05 μm) are capable of permeating cell membranes and have higher bioavailability compared to micro-sized microbeads (0.5 and 6 μm), resulting in reduced growth rates and reproduction in response to cellular damage.

Although the adverse effects of microplastics on marine organisms have been widely studied, their effects at the molecular level are poorly understood. The toxicity of nanoparticles in cells mainly arises from oxidative stress via the generation of ROS. Accumulated ROS subsequently elicits several biological responses such as oxidative stress-induced signaling pathways, apoptosis, and inflammation^39^,^40^,^41^. In this study, we focused mainly on oxidative stress-induced MAPK pathways and Nrf2 when examining upstream regulatory pathways of the antioxidant defense system in response to microplastic exposure. MAPKs consisted of ERK, JNK, and p38, which are activated by ROS, resulting in cellular physiology effects^42^,^43^,^44^. ERK signaling pathways are involved in cell survival and proliferation in response to oxidative stress, whereas JNK and P38 are commonly related to cell death processes^45^,^46^,^47^. In addition to MAPK, Nrf2 is considered a key regulatory transcription factor for genes encoding defensive enzymes including GR, GPX, and SOD in response to oxidative stress^48^,^49^,^50^. In our study, ROS was significantly induced in response to 0.05-μm microbeads, indicating that was positively correlated with expression of p-ERK, p-p38, and Nrf2. Significant induction of ROS (*P* < 0.05) in 0.05-μm microbead-exposed *P. nana* and size-dependent expression of p-ERK, p-p38, and Nrf2 supported *in vivo* endpoints through ingestion/egestion experiments. In *P. nana*, a prolonged retention of 0.05-μm microbeads is likely to cause by greater induced oxidative stress compared to that caused by larger microbeads. With co-administration of a ROS scavenger NAC, ROS levels and activation of p-ERK, p-p38, and Nrf2 were not observed, suggesting that microplastic-induced oxidative stress could be considered a trigger of oxidative stress-dependent signaling pathways mediated by p-p38, p-ERK, and Nrf2. Activation of Nrf2 is closely associated with MAPK phosphorylation as a downstream transcription factor. In human hepatoma cells, ERK and JNK pathways played a regulatory role in Nrf2 activation, while the p38 pathway plays a negative role^51^. In human epithelial cells, cerium oxide nanoparticles induce oxidative stress with activation of heme oxygenase-1 via the p38/Nrf2 signaling pathway^52^. Taken together, in *P. nana*, ERK and p38 were activated in response to microplastic-induced oxidative stresses. Although the precise regulatory mechanisms between MAPK pathways and Nrf2 activation have not been determined, our results suggest that activation of the p38 and ERK pathways in *P. nana* involve the defense mechanism against microplastic-induced oxidative stress via the MAPK/Nrf2 pathway.

Following Nrf2 activation, antioxidant enzymatic activity was investigated in response to microbeads exposure. In our study, enzymatic activity and GSH content were increased in a size-dependent manner in response to microbead exposure (Fig. S1). GSH-related antioxidant enzymes are a key modulator of the phase II detoxification mechanism against oxidative stress due to their redox capacity related to GSH molecules^53^. In aquatic invertebrates, antioxidant enzyme activities increases in response to various stressors causing oxidative stress including UV-B radiation^54^, metal^55^, and nanoparticle exposure^56^, suggesting that these enzymes play an important role in detoxifying oxidative stresses. In the case of GSH content, it was controversial as shown in the microplastic-exposed rotifer *B. koreanus*. In *B. koreanus*, GSH contents had a slightly decreased pattern, while the activity of GPx, GR, SOD, and GST was increased in a microbead size-dependent manner^12^. However, considering the multifunctional roles of GSH molecules in various biological processes such as DNA synthesis, microtubular-related processes, and immune function, GSH content may vary depending on the species or other experimental conditions^57^,^58^,^59^. Taken together, in *P. nana*, the activation of GSH-related antioxidant enzymes was involved in microplastic-induced oxidative stress, implying that these processes are likely to be an important defense mechanism against microplastic-induced oxidative stress in *P. nana*.

**Figure 6:**
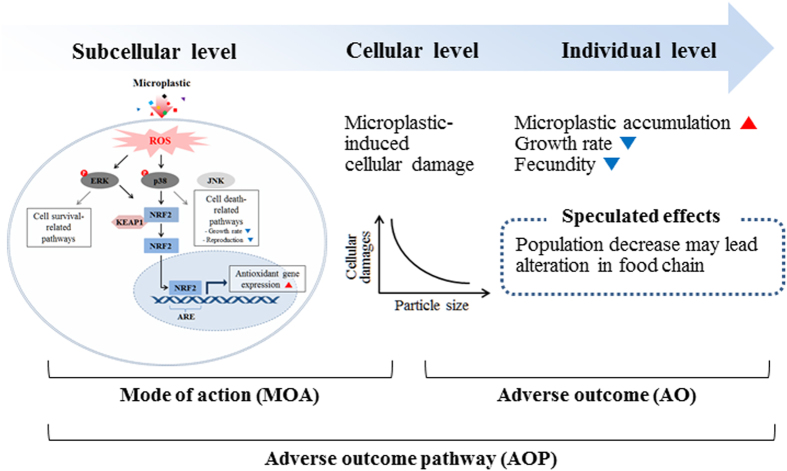
Proposed AOPs for microplastic exposure in the marine copepod *P. nana*.

As a summary of the AOP in microplastic-exposed *P. nana* (Fig. 6), nano-sized polystyrene microbeads (0.05 μm) can permeate cell membranes with higher bioavailability and can generate stronger oxidative stress compared to micro-sized microbeads (0.5 and 6 μm). Penetration of the cell membrane by microbeads and generation of oxidative stress can cause cellular damage and activate signal transduction, leading to size-dependent oxidative stress responses that reduce growth rate and reproduction as a consequence of cellular damage. In addition to adverse effects at the individual level, potential effects at the ecosystem population level would be expected as the *P. nana* copepod population is known to play important roles in the aquatic ecosystem^28^. Besides the negative effects of microplastics which have been clearly demonstrated in this study, further studies with field-based concentration of nano-sized microplastics are necessary to better understand comprehensive impacts on the marine ecosystem, since the information on environmental concentration of nano-sized microplastics is still unknown. In conclusion, our results demonstrated adverse effects related to size-dependent toxicity in response to ingestion/egestion of microplastics in the marine copepod *P. nana*. Also, this study is the first insight into the mode of action of microplastics with the AOP in the marine copepod.

## Materials and Methods

### Copepod culture

The copepod *P. nana* was maintained under controlled incubator conditions with a 12 h light/12 h dark cycle at a temperature of 25 °C in artificial sea water (ASW). The salinity of the culture medium was 15 practical salinity units (psu) with a pH 8.0. *P. nana* was fed a diet of *Chlorella* sp. (approximately 6 × 10^4^ cells/mL) once per day. The identity of copepod species used for this experiment was verified based on morphological characteristics and sequence analysis of *P. nana* mitochondrial DNA cytochrome oxidase 1 (*CO1*) as the barcoding gene for the animal^60^.

### Polystyrene microbeads

Non-functionalized polystyrene microbeads of 0.05-, 0.5-, and 6-μm diameters were purchased from Polyscience (Warrington, PA, USA). Polystyrene was chosen, as it is one of the most abundant polymers in marine debris^1^. In this study, 6-μm microbeads were considered as a model microplastic, as it is similar in size to *Chlorella* sp. which was supplied as a diet during the experiments. As plastics are degraded into smaller fragments^61^,^62^,^63^, 0.05-and 0.5-μm microbeads were chosen to investigate the size-dependent toxicity of the microplastic in *P. nana*. 0.05-μm diameters of polystyrene microbeads were used as nano-sized microplastics, whereas 0.5- and 6-μm diameters were used as micro-sized microplastics following the definition made by Klaine *et al*.^64^. For ingestion experiments, the same diameters of fluorescently labeled polystyrene microbeads (excitation at 441 nm/emission at 486 nm; Warrington, PA, USA) were used. The quantitative relationship between the mass concentration and the number of particles (particles/mL) of the microbeads was shown in Table 1.

### Polystyrene microbeads ingestion and egestion

To visualize the ingestion of microplastics in *P. nana, P. nana* was exposed to 10 μg/mL of different sizes (0.05, 0.5, and 6 μm) of fluorescently labeled polystyrene microbeads (approximately 100 individuals in 50 mL ASW), and then incubated in the dark for 24 h. Copepods were then washed by ASW and fixed with 4% formaldehyde. Accumulated fluorescently labeled microbeads in *P. nana* were observed by a fluorescent microscope (Olympus IX71, Olympus Corporation; Tokyo, Japan).

For the egestion study of microplastics, different sizes of fluorescently labeled microbeads (10 μg/mL)-exposed *P. nana* exposed were transferred in clean ASW. After 24-h incubation in the clean ASW, *P. nana* were washed in clean ASW and fixed in 4% formaldehyde. Fluorescently labeled microbeads remaining in *P. nana* were then observed using a fluorescent microscope.

### *In vivo* toxicity tests

Developmental time and fecundity were measured using *P. nana* exposed to 0.05-, 0.5-, and 6-μm diameters of polystyrene microbeads at 0.1, 1, 10, and 20 μg/mL concentrations to investigate the size- and concentration-dependent toxicity of microplastics in *P. nana*.

To examine the retardation in developmental time, ovigerous females were initially collected and incubated in ASW for 2 h. Newborn nauplii were then collected for *in vivo* experiments. Ten nauplii were transferred into a 12 well culture plate (SPL, Seoul, South Korea) with 4 mL of ASW containing 0.05-, 0.5-, and 6-μm microbeads at concentrations of 0.1, 1, 10, and 20 μg/mL. Development stages of nauplii in each exposure groups were observed under a stereomicroscope (SZX-ILLK200, Olympus Corporation, Tokyo, Japan) every 24 h. Observation was finished when all nauplii had matured, while observations on ovigerous females were continued to measure fecundity.

To examine the effects of microplastic exposure on reproduction in *P. nana*, 10 individual ovigerous females were transferred to 12-well culture plates and exposed to 0.1, 1, 10, and 20 μg/mL concentrations of different sizes of microbeads in triplicate. Fecundity was measured by counting newborn nauplii every 24 h.

During the experiments, half of the testing media was renewed daily with green algae *Chlorella* sp. to maintain the dietary concentration. All experiments were performed in triplicate and the temperature was maintained at 25 °C.

### Measurement of the level of reactive oxygen species

To examine whether polystyrene microbeads induce oxidative stress in the copepod *P. nana*, intracellular ROS levels were measured using 2′7′-dichlorodihydrofluorescein diacetate (H_2_DCFDA; Molecular Probes, Eugene, OR, USA), which was oxidized to fluorescent dichlorofluorescein (DCF) by intracellular ROS. After exposure of different concentrations (0.1, 1, 10, and 20 μg/ml) of microbeads for 24 h (approximately 500 individuals in 250 mL ASW), copepods were homogenized in a buffer containing 0.32 M sucrose, 20 mM HEPES, 1 mM MgCl_2_ and 0.5 mM PMSF (pH 7.4) with a Teflon homogenizer. The homogenate was centrifuged at 10,000xg for 20 min at 4 °C. The supernatant was then collected for measurements. A 96-well black plates was filled with 170 μL phosphate-buffered saline (PBS) buffer, 10 μL probe (H_2_DCFDA at a final concentration of 40 μM), and 20 μL supernatant fraction to a final volume (200 μL). Measurements were obtained with an excitation wavelength at 485 nm and emission wavelength at 520 nm with a fluorescence spectroscope. ROS measurements were normalized by total protein and were represented as a percentage of DCF fluorescence. Total protein was determined via the Bradford method^65^.

### Antibodies

Polyclonal antibodies to phospho-SAPK/JNK (anti-rabbit, Thr-183/Tyr-185), ERK (anti-rabbit), and JNK (anti-rabbit) were obtained from Cell Signaling Technology (Beverly, MA, USA). Polyclonal antibodies to p38 MAPK (anti-rabbit), phospho-ERK1/2 (anti-mouse, Thr-202/Tyr-204), phospho-p38 (anti-rabbit, Tyr-182), and Nrf2 (anti-rabbit) were purchased from Santa Cruz Biotechnology (Santa Cruz, CA, USA). Monoclonal antibodies to tubulin were obtained from Sigma-Aldrich (St. Louis, MO, USA). Anti-mouse Alexa Fluor 488, and anti-rabbit Alexa Fluor 488 were purchased from Invitrogen (Carlsbad, CA, USA).

### Western blot analysis

*P. nana* (approximately 500 individuals in 250 mL ASW) were exposed to different sizes (0, 0.05, 0.5, and 6 μm) of microbeads (concentration 10 μg/ml) for 24 h, and whole *P. nana* were homogenized to extract protein in lysis buffer (40 mM Tris–HCl [pH 8.0], 120 mM NaCl, 0.1% Nonidet-P40) containing a 1 × EDTA-free complete protease inhibitor cocktail (Roche, South San Francisco, CA, USA; 1 mM PMSF, 10 mM NaF, 10 mM NaN_3_, 10 mM NaPPi, 10 mM, β-glycerophosphate, 10 mM *p*-nitrophenylphosphate, 0.5 mM benzamidine, HCl, 1.5 mM Na_3_VO_4_, and 20 μg/ml heparin). Protein was separated using 10% sodium dodecyl sulfate-polyacrylamide gel electrophoresis (SDS-PAGE) and transferred to a nitrocellulose membrane (Amersham, Arlington Heights, IL, USA). The membrane was blocked with 2.5% bovine serum albumin (BSA) in Tris-buffered saline and incubated with primary antibodies (1:1,000) overnight at 4 °C. The blots were developed using a peroxidase-conjugated secondary antibody (1:1,000), and proteins were visualized using enhanced chemiluminescence procedures (Amersham) according to the manufacturer’s protocol.

### Treatment with ROS scavenger N-acetyl-L-cysteine (NAC)

To determine whether ROS is the main trigger of MAPK phosphorylation, *P. nana* were exposed to different sizes (0-, 0.05-, 0.5-, and 6-μm) of microbeads in the presence of NAC. Total protein was extracted and followed the same Western blot procedure described above.

### Measurement of GSH-related enzyme activity

The glutathione reductase (GR) and glutathione peroxidase (GPx) activity was measured via an enzymatic method using GR and GPx cellular activity assay kits, respectively (Sigma-Aldrich). After 0.05-, 0.5-, and 6-μm diameters of polystyrene microbeads exposure at 10 μg/mL for 24 h, *P. nana* (approximately 500 individuals in 250 mL ASW) were homogenized in cold buffer (50 mM Tris–HCl, 5 mM EDTA, and 1 mM 2-mercaptoethanol, pH 7.5) at a ratio of 1–4 (w/v) with a Teflon homogenizer. The homogenates were centrifuged at 10,000 × g for 10 min at 4 °C. The upper aqueous layer containing the enzyme was collected for enzymatic assay according to the manufacturer’s protocol. The GPx and GR activity was then measured at an absorbance of 340 nm with a spectrophotometer at 25 °C.

In the case of GST, *P. nana* were homogenized in cold buffer (0.25 M sucrose, 10 mM Tris, 1 mM EDTA, 0.2 mM DTT and 0.1 mM PMSF, pH 7.4) at a ratio of 1–4 (w/v) with a Teflon homogenizer. The homogenates were centrifuged at 10,000× g for 10 min at 4 °C. The cytosolic fraction containing the enzyme was collected for an enzymatic assay with 1-chloro-2,4-dinitrobenzene (CDNB) as a substrate. The enzymatic assay monitored the conjugation of CDNB and GSH at 340 nm with a spectrophotometer at 25 °C.

The total SOD activity was measured via an enzymatic method using a SOD assay kit (Sigma-Aldrich). After exposure to different diameters of microbeads at 20 μg/mL, the copepods were homogenized in an ice-cold buffer (0.25 M sucrose, 0.5% Triton X-100, pH 7.5) at a ratio of 1–4 (w/v) with a Teflon homogenizer. The homogenates were centrifuged at 3,000 × g for 10 min at 4 °C. The upper aqueous layer containing the enzyme was collected for the enzymatic assay according to the manufacturer’s protocol. The total SOD activity was then measured at an absorbance level of 440 nm using a spectrophotometer at 25 °C. Enzymatic activity was normalized by total protein and presented as % of control. Total protein was determined using the Bradford method^65^.

### Statistical analysis

SPSS ver. 17.0 (SPSS Inc.; Chicago, IL, USA) was used for statistical analysis. Data are expressed as mean ± S.D. Significant differences between control and the exposed groups were analyzed using the Student’s paired t-test and one-way and/or multiple-comparison ANOVA followed by Tukey’s test. *P* < 0.05 was considered significant.

## Additional Information

**How to cite this article**: Jeong, C.-B. *et al*. Adverse effects of microplastics and oxidative stress-induced MAPK/Nrf2 pathway-mediated defense mechanisms in the marine copepod *Paracyclopina nana. Sci. Rep.* **7**, 41323; doi: 10.1038/srep41323 (2017).

**Publisher's note:** Springer Nature remains neutral with regard to jurisdictional claims in published maps and institutional affiliations.

## Supplementary Material

Supplementary Figure S1

## Figures and Tables

**Table 1:** The quantitative relationship between the mass concentration and the number of particles (particles/mL) of the microbeads used in this study.

Weight (μg/mL)	0.05	0.5	6 (μm)
0.1	1.46 × 10^9^	1.46 × 10^6^	8.4 × 10^2^
1	1.46 × 10^10^	1.46 × 10^7^	8.4 × 10^3^
10	1.46 × 10^11^	1.46 × 10^8^	8.4 × 10^4^
20	2.91 × 10^11^	2.91 × 10^8^	1.68 × 10^5^
