# Supplementary material for: Adverse effects of microplastics and oxidative stress-induced MAPK/Nrf2 pathway-mediated defense mechanisms in the marine copepod Paracyclopina nana
Source: Sci Rep. 2017 Jan 24;7:41323. doi: 10.1038/srep41323 (PMC5259799; doi:10.1038/srep41323)
Supplement: Supplementary Figure S1 [file srep41323-s1.pdf]

Supporting Information

Adverse effects of microplastics and oxidative stress-induced MAPK/Nrf2 pathway-mediated defense mechanisms in the marine copepod *Paracyclopina nana*

Chang-Bum Jeong[1,2,#], Hye-Min Kang[1,#], Min-Chul Lee[1], Duck-Hyun Kim[1], Jeonghoon Han[1], Dae-Sik Hwang[1], Sami Souissi[3], Su-Jae Lee[4], Kyung-Hoon Shin[5], Heum Gi Park[6,*], and Jae-Seong Lee[1,*]

[1]*Department of Biological Science, College of Science, Sungkyunkwan University, Suwon 16419, South Korea*

[2]*Department of Chemistry, College of Natural Sciences, Hanyang University, Seoul 04763, South Korea*

[3]*Univ. Lille, CNRS, Univ. Littoral Cote d'Opale, UMR 8187, LOG, Laboratoire d'Océanologie et de Géosciences, F 62930 Wimereux, France*

[4]*Department of Life Sciences, College of Natural Sciences, Hanyang University, Seoul 04763, South Korea*

[5]*Department of Marine Sciences and Convergent Technology, College of Science & Technology, Hanyang University, Ansan 15588, South Korea*

[6]*Department of Marine Resource Development, College of Life Sciences, Gangneung-Wonju National University, Gangneung 25457, South Korea*

[#]These authors equally contributed to this work.

[*]Corresponding authors. E-mail: jslee2@skku.edu (J.-S. Lee) or hgpark@gwnu.ac.kr (H.G. Park)

SUPPLEMENTAL MATERIAL, FIGURE

A. Supplementary Figures

Fig. S1

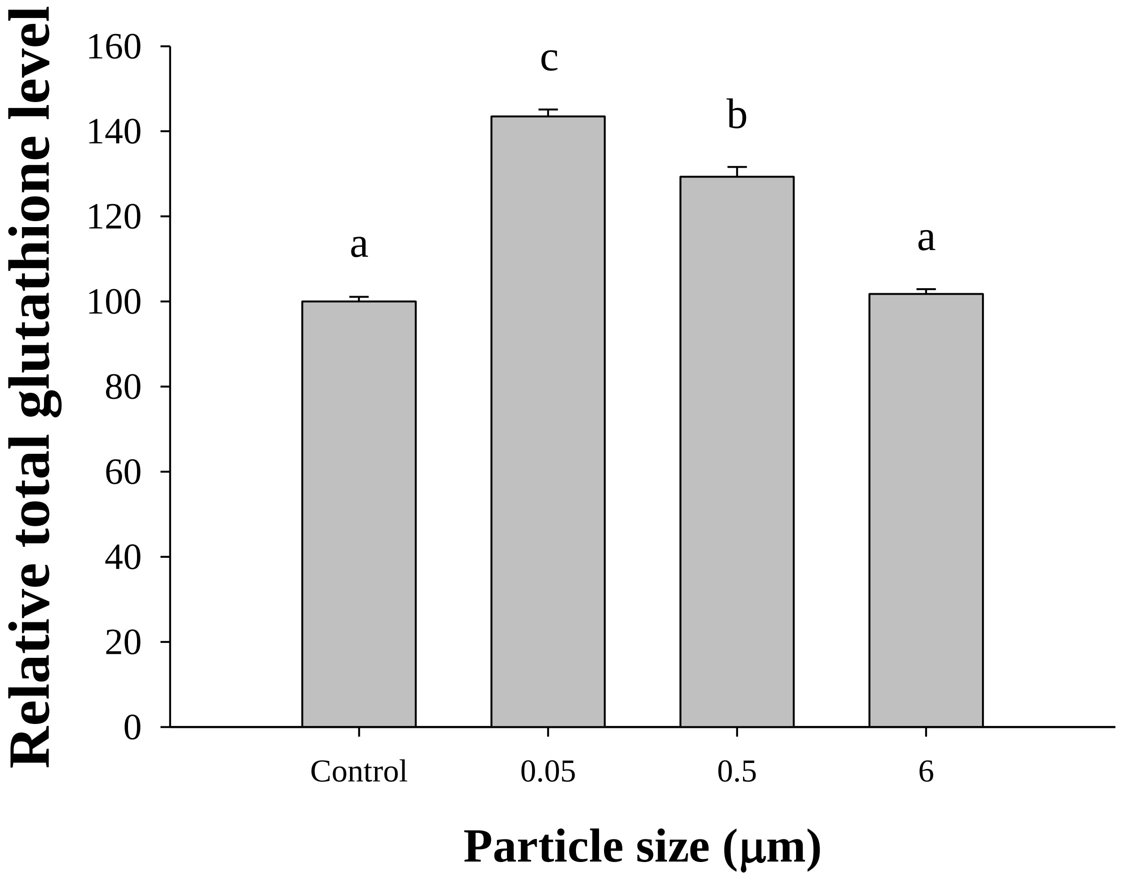

B. Supplementary figure legends

Fig. S1 Effects of exposure to polystyrene microbeads of different diameters (0.05, 0.5, and 6 µm) on total glutathione level. All polystyrene microbeads were used at 20 µg/ml. Enzymatic activities are represented as % of controls. Different letters above columns indicate significant differences, defined as $P < 0.05$.
